# Development of skin diseases following systemic exposure: example of dioxins

**DOI:** 10.3389/ftox.2023.1243192

**Published:** 2023-08-28

**Authors:** Olivier Sorg, Jean-Hilaire Saurat

**Affiliations:** Clinical Pharmacology and Toxicology, University of Geneva, Geneva, Switzerland

**Keywords:** dioxin, skin, toxicology, AhR, biomarkers, ingestion

## Abstract

Most skin manifestations of exposure to toxic compounds are a consequence of a direct contact with the toxicants. However, some toxicants may reach the skin following systemic exposure, and promote skin diseases. Good examples of such chemicals are dioxin-like compounds. This family of lipophilic molecules comprises polychlorinated (dibenzodioxins, dibenzofurans and biphenyls). The most potent member of this family is 2,3,7,8-tetrachlorodibenzo-*p*-dioxin (TCDD). Following oral ingestion of as little as a few mg TCDD, skin lesions appear in a couple of weeks, starting from the face and diffuse then on the trunk and limbs. This syndrome was historically called “chloracne” and the skin lesions have now been shown to be skin hamartoma induced by TCDD. Sebaceous glands release their lipid content on the surface of the skin by a holocrine secretion, and so any lost sebocyte should be transmitted to progenitor cells to differentiate and migrate to the sebaceous gland to replace the lost sebocyte. TCDD acts by inducing a switch in this signal and skin hamartoma develop in place of new sebocytes.

## Introduction

**FIGURE 1:**
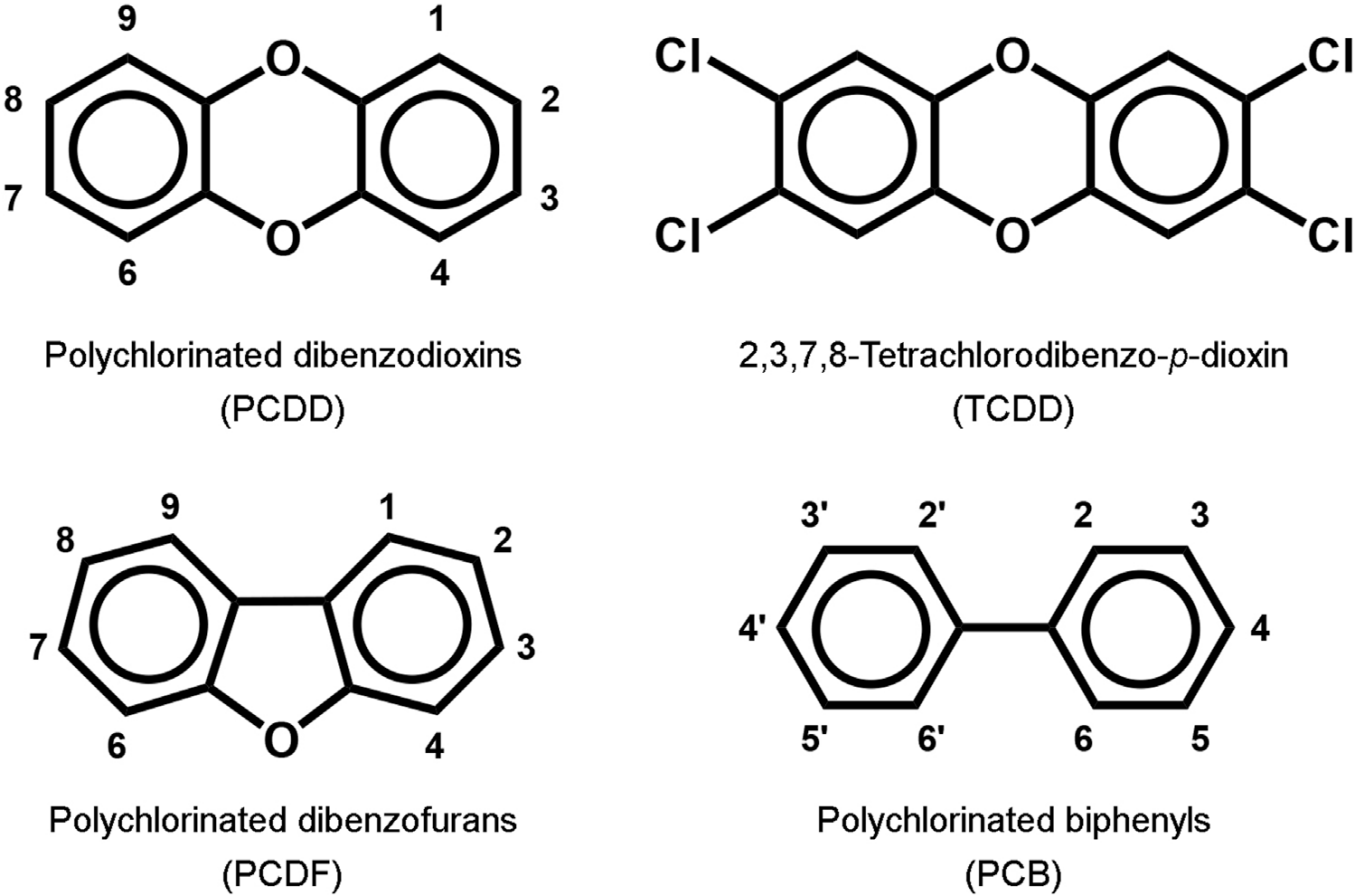
Chemical structures of dioxin-like compounds.

Persistent organic pollutants collectively referred to as dioxin-like compounds include polyhalogenated aromatic hydrocarbons such as polychlorinated dibenzodioxins (PCDD), polychlorinated dibenzofurans (PCDF) and polychlorinated biphenyls (PCB) (Figure 1). They were involved in various industrial accidents in the 20th century such as the BASF plant in Ludwigshafen in 1953 (Thiess et al., 1982) and the Icmesa factory in Seveso in 1976 (Assennato et al., 1989). 2,3,7,8-Tetrachlorodibenzo-*p*-dioxin (TCDD), the most biologically potent of this family, was shown to be the toxic contaminant of the Agent Orange used by the U.S. Army during the Vietnam War in the 1960s (Steele et al., 1990). During the Ukrainian presidential campaign in 2004, the candidate Victor Yushchenko was poisoned by TCDD at a dinner party organised by the Ukrainian security services (McKee, 2009; Sorg et al., 2009; Saurat et al., 2012). The members of the dioxin-like family are not volatile, and all reported intoxications by these compounds were due to ingestion of contaminated materials. However, the hallmark of clinical manifestations following oral exposure to dioxins is a cutaneous syndrome known as chloracne (Panteleyev and Bickers, 2006; Saurat et al., 2012), which illustrates a mechanism of skin toxicity promoted by chemicals that reached the skin via oral route, and not by direct contact to the skin.

## Sebaceous gland homeostasis

Sebaceous glands are skin appendages that produce various lipids forming a thin protective film at the skin surface (Schneider and Paus, 2010). Terminally differentiated sebocytes are filled with lipids, undergo nuclear degradation and membrane lysis, and finally release their content by holocrine secretion (the sebum), which flows to the surface of the skin. To maintain a physiological renewal of sebaceous glands, the lost sebocytes must be replaced.

During the terminal differentiation of sebocytes, a signal should be released to induce pluripotent stem cells found in the hair follicle junctional zone to differentiate and migrate to the outermost layer of sebaceous glands. Different proteins, such as SRY-box transcription factor 9 (SOX9), leucine-rich repeats and immunoglobulin-like domains protein 1 (LRIG1) and (leucine-rich repeat-containing G-protein-coupled receptor 6 (LGR6), are expressed in sebocyte progenitor cells (Clayton et al., 2019; Geueke and Niemann, 2021), and might be involved in the signalling pathway for sebaceous gland renewal.

In cultured sebocytes, a downregulation of LRIG1 by siRNA significantly decreased the CYP1A1 response to TCDD, indicating that LRIG1 contributes to a higher susceptibility of AhR agonists (Fontao et al., 2017). Studies in mice showed that following intraperitoneal injection by TCDD or β-naphthoflavone—another AhR agonist—the first cutaneous targets were sebaceous glands. When these mice were treated by topical application of TCDD, LRIG1-bearing pluripotent cells found in the vicinity of sebaceous glands showed a strong activation of CYP1A1, thus appearing as a main target of TCDD that may be involved in the disturbed turnover of sebaceous gland and hamartoma formation (Fontao et al., 2017).

According to recent studies TCDD, a very lipophilic compound, may accumulate in sebum and leads to deregulation of sebocyte differentiation via i) the activation of the transcriptional repressor B lymphocyte maturation protein 1 (Blimp1), as well as ii) the stimulation of the proliferation of interfollicular epidermis mediated by the Wnt signalling pathway. This may explain the observed atrophy of sebaceous glands and the formation of the mentioned hamartoma (MADISH), the hallmark of TCDD cutaneous toxicity (Ikuta et al., 2010; Bock, 2017a; Bock, 2017b; Feldman et al., 2019).

## Biology of dioxins

Dioxin-like compounds are very lipophilic and may cross the plasma membrane of cells via simple diffusion. Once in the cytosol, they bind to an intracellular receptor, the aryl hydrocarbon receptor (AhR). Following AhR binding by an agonist, chaperone proteins maintaining AhR in a quiescent form are released, the (agonist-AhR) complex recruits another protein—the AhR-nuclear-translocator (ARNT)—then the new complex is translocated to the nucleus and binds to xenobiotic response elements (XRE) in the promoters of various genes, leading to the modulation of their expression (Abel and Haarmann-Stemmann, 2010; Guyot et al., 2013; Sorg, 2014).

Many genes have XREs on their promoter. Moreover, AhR interacts with different signalling pathways, and may cross-talk with other nuclear receptors. This may explain the pleiotropic actions promoted by the structural diversity of its agonists (Hahn, 2002; Tijet et al., 2006; Borlak and Jenke, 2008; Furness and Whelan, 2009; Hayes et al., 2009; Ma et al., 2009; Puga et al., 2009; Ohtake et al., 2011; Guyot et al., 2013), and makes AhR a promising therapeutic target for chronic inflammatory skin diseases (Kim et al., 2022).

TCDD toxicity is characterised by a very large range of sensitivities among mammals, the LD_50_ for Syrian hamsters (≈10 mg/kg) being 10,000 times higher than that of guinea pigs (≈1 μg/kg) (Geyer et al., 1993). Following oral lethal doses of TCDD animal die mainly from liver failure and gastrointestinal haemorrhage (Niittynen et al., 2007). The gastrointestinal tract is the first target, followed by the liver and the pancreas, then the peripheral nervous system, and finally the skin, which is also the last organ to recover (Saurat et al., 2012).

Dioxin-like compounds are very lipophilic and are found almost exclusively in body fat; this allows to determine the body burden of dioxin from the blood value of dioxin expressed as [pg/g lipid weight (lw)] and the body fat content (Sorg et al., 2009).

Depending on their reactivity toward the phase I and II enzymes catalysing their metabolic conversion, they stay in subcutaneous fat for various periods of time, where they are delivered to the skin regularly and induce a disruption of cutaneous biology. This is particularly the case for TCDD, which has a very long biological half-life (7–10 years) in spite of a huge activation of cytochromes P450 1A1, 1A2 and 1B1; indeed, in the case of Victor Yushchenko, only trace amounts of two hydroxylated metabolites of TCDD were found several months following TCDD exposure, corresponding to the peak of cutaneous symptoms (Sorg et al., 2009; Saurat et al., 2012). CYP1A1 is highly induced following AhR-dependent signalling pathway, and is a valuable biomarker of exposure to any dioxin-like compound (Chessa et al., 2021).

Various AhR-dependent cutaneous effects were reported in animal or *in vitro* models. *In utero* exposure of mice to TCDD accelerated epidermal barrier formation and promoted a transient chloracne-like syndrome with sebaceous gland hypoplasia and cyst formation in their offspring (Bhuju et al., 2021). Peripheral blood mononuclear cells and CD4^+^ T cells from patients with psoriasis or atopic dermatitis were more sensitive to the AhR-dependent response to TCDD than these cells from healthy volunteers. These effects were not observed with the AhR agonist N-formylindolo[3,2-b]carbazole (FICZ), which has a much shorter biological half-life than TCDD (Um et al., 2020). Retinoids, in particular isotretinoin, are used to treat acneiform lesions; however, although their metabolism is impaired by dioxins (Esteban et al., 2021), retinoids failed to reverse TCDD-induced cutaneous toxicity, as demonstrated by various *in vitro* and animal models (Rudyak et al., 2018). An aqueous extract of *Deschampsia antarctica* protected cultured human keratinocytes and fibroblasts from TCDD-induced toxicity, which might be interesting to treat chloracne-like syndromes if confirmed in clinical trials (Zamarron et al., 2019).

## The case of Victor Yushchenko

**FIGURE 2:**
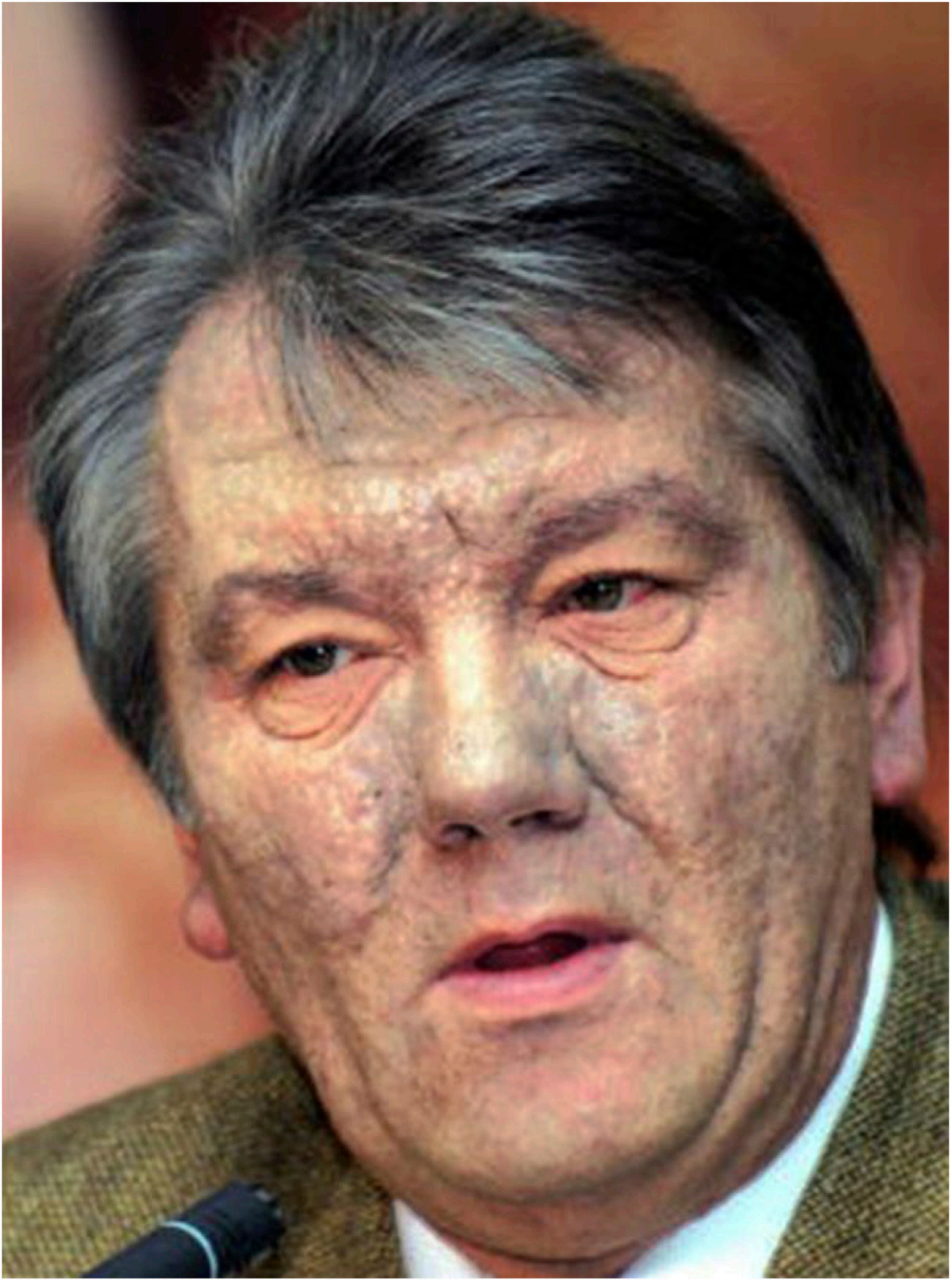
Victor Yushchenko several weeks after being exposed to TCDD by ingestion.

In late September 2004, Victor Yushchenko, a candidate for the Ukraine presidency, appeared disfigured in his meetings and on TV channels. His face was swollen and had many skin lesions of greyish colour (Figure 2). A few weeks earlier, coming back from a dinner organised by senior officials of the Ukrainian security services, he became seriously ill. Victor Yushchenko was hospitalised at a private clinic in Vienna for 9 days. He was diagnosed with acute pancreatitis of unknown origin, associated with ulcerations of the intestine and enlargement of the liver.

In December, a sample of his blood sent to a lab in Amsterdam appeared to be contaminated by dioxin-like compounds that activate the AhR signalling pathway, and in January 2005, a chemical analysis performed in Dübendorf (Switzerland) revealed a blood concentration of 110 ng TCDD/g (lw), i.e., more than 5,000 times higher than the global TCDD equivalent of toxicity (TEQ) found in the general population, and corresponding to a body dose of about 2 mg TCDD. It was clear that during his dinner on 5 September 2004, it had been poisoned by TCDD (McKee, 2009; Sorg et al., 2009; Ryan, 2011).

The cutaneous signs appeared 2–3 weeks later, and amplified on a period of 18 months, before decreasing slowly up to 3.5 years after poisoning. The hallmark of the cutaneous signs of dioxin toxicity, known as chloracne, is the development of particular skin lesions showing both “loss of structure” and “gain of structure” features, which we identified as hamartomas and named “metabolising acquired dioxin-induced skin hamartomas” (MADISH) (Saurat et al., 2012). A whole-genome transcriptional analysis of skin samples of V. Yushchenko taken 5 months after TCDD poisoning, compared to matching controls, identified 329 genes with more than four-fold upregulation and 1,533 genes with more than four-fold downregulation. Genes coding for enzymes involved in the metabolism of xenobiotics, as well as genes involved in inflammation and extracellular matrix modulation, were highly induced, whereas many genes of the metabolism of lipids, in particular for the sebogenesis, were highly repressed (Saurat et al., 2012).

Parallel to the development of these hamartomas, there was a disappearance of all sebaceous glands. Since the sebocytes are lost during the holocrine secretion of their content, it seems that TCDD promoted a disruption of the signalling pathway that normally induces the renewal of sebocytes from progenitor cells. Instead, new structures, the mentioned hamartomas, are produced and sebaceous glands degenerate.

## Chloracne-like syndromes

The skin manifestations observed following dioxin exposure seem not specific of these compounds. Besides the herbicides and defoliants 2,4-dichlorophenoxyacetic acid and 2,4,5-trichlorophenoxyacetic acid, which constituted the Agent Orange used by the US Army during the Vietnam War and contained TCDD as impurity, other chlorinated compounds used in the synthesis of herbicides, such as 3,3′,4,4′-tetrachloroazobenzene and 3,3′,4,4′-tetrachloroazoxybenzene, were shown to produce chloracne-like skin lesions in chemical workers (Kimbrough, 1980; Scarisbrick and Martin, 1981).

Gawkrodger et al. (2009) reported a case series of seven organic chemists exposed to novel polycyclic halogenated chemicals of the family of triazoloquinoxalines, who developed clinical skin manifestations similar to chloracne, without any other signs of toxicity.

Passarini et al. (2010) reported a case series of nine patients with clinical signs of chloracne. Histological analyses of skin lesions from these patients revealed structures very similar to those observed in the skin samples from Yushchenko. However, the standard chemical analysis of dioxin-like compounds, including 7 PCDD, 10 PCDF and 8 PCB, in the blood of these patients did not show any values outside the normal range of the general population. The cause of these clinical symptoms was not identified.

In a more general manner, many dermatologists encounter patients living near dumping sites and landfills who developed skin lesions similar to MADISH (Megna et al., 2017). These patients were exposed regularly to various mixtures of chemicals, so it is quite difficult to identify the substances involved in the observed skin lesions.

All these examples of chloracne-like skin manifestations raise the question of the biochemical mechanism involved in the development of these particular skin lesions. Most known chloracnegens activate the AhR-dependent signalling pathway. However, various cases of chloracne-like syndromes, such as the case series of Passarini and colleagues mentioned above, are note related to the exposure of AhR agonists. On the other hand, all AhR agonists do not promote chloracne (Forrester et al., 2014). In particular high levels of AhR agonist activity may be reached in the blood following the consumption of various vegetables of the family of brassicaceae, and people who consume these vegetables regularly do not develop skin lesions (Connor et al., 2008).

## Other skin diseases following systemic exposure

Besides dioxins other compounds may reach the skin via the blood circulation and exert a toxicity to the skin. Dysregulation of hormone homeostasis by endocrine disruptors may affect the skin too (dioxins are only an example) and promotes various skin conditions via the binding of cellular targets in keratinocytes, melanocytes, sebocytes and immune skin cells (Ju and Zouboulis, 2016). Ingestion of thallium, a rodenticide no longer in use, induces skin lesions resulting in parakeratotic keratosis, the degeneration of keratinocytes and finally an ulceration of the skin (Bruner, 1991). Toxic epidermal necrolysis is another example of a specific cutaneous toxicity following ingestion of chemically unrelated compounds targeting the death receptor Fas (CD95), and resulting in the detachment of the epidermis from the dermis (Viard et al., 1998; Harr and French, 2010). Systemic contact dermatitis develops in people previously exposed to an allergen by direct contact to the skin when they are exposed again to this allergen via the blood circulation (Park et al., 2021).

## Discussion

In the present review we gave an example of a family of compounds that promote a specific disease of the skin following enteral exposure. In the case of dioxins, the main known activated signalling pathway induces the transcription of genes involved in their metabolism and elimination; however, this mechanism does not function for TCDD, the most potent member of this family, because this compound resists to the metabolism by the phase I enzymes it induces, which confers to TCDD a very long biological half-life responsible for its cutaneous toxicity. To better understand the mode of action of TCDD and other chloracnegens we have to find other mechanisms of action besides the activation of xenobiotic response elements found in the promoter regions of many genes. The binding of such compounds to AhR may trigger cross-talks with other signalling pathways, as already mentioned (Hahn, 2002; Tijet et al., 2006; Borlak and Jenke, 2008; Furness and Whelan, 2009; Hayes et al., 2009; Ma et al., 2009; Puga et al., 2009; Ohtake et al., 2011; Guyot et al., 2013), although it is not clear how this pleiotropic action of AhR may induce the mentioned clinical manifestations.

As a take-home message, it is important to bear in mind that skin chemicals entering the body via systemic exposure may reach the skin and induce a cutaneous toxicity. In general toxicology the skin is often overlooked and not considered as a possible target organ, whatever the mode of exposure. Most often the skin is only seen as a possible route of entry for compounds that may be toxic for internal organs.

## References

[B1] Abel J. Haarmann-Stemmann T. (2010). An introduction to the molecular basics of aryl hydrocarbon receptor biology. Biol. Chem. 391, 1235–1248. 10.1515/BC.2010.128 20868221

[B2] Assennato G. Cervino D. Emmett E. A. Longo G. Merlo F. (1989). Follow-up of subjects who developed chloracne following TCDD exposure at Seveso. Am. J. Ind. Med. 16, 119–125. 10.1002/ajim.4700160203 2773943

[B3] Bhuju J. Olesen K. M. Muenyi C. S. Patel T. S. Read R. W. Thompson L. (2021). Cutaneous effects of in utero and lactational exposure of C57bl/6J mice to 2. Tetrachlorodibenzo-p-dioxin 3 (7), 8. 10.3390/toxics9080192 PMC8402454 34437510

[B4] Bock K. W. (2017a). 2,3,7,8-Tetrachlorodibenzo-p-dioxin (TCDD)-mediated deregulation of myeloid and sebaceous gland stem/progenitor cell homeostasis. Arch. Toxicol. 91, 2295–2301. 10.1007/s00204-017-1965-2 28386637

[B5] Bock K. W. (2017b). From dioxin toxicity to putative physiologic functions of the human Ah receptor in homeostasis of stem/progenitor cells. Biochem. Pharmacol. 123, 1–7. 10.1016/j.bcp.2016.06.015 27349986

[B6] Borlak J. Jenke H. S. (2008). Cross-talk between aryl hydrocarbon receptor and mitogen-activated protein kinase signaling pathway in liver cancer through c-raf transcriptional regulation. Mol. Cancer Res. 6, 1326–1336. 10.1158/1541-7786.MCR-08-0042 18708364

[B7] Bruner R. H. (1991). Pathological processes of skin Damage related to toxicant exposure, Hobson D. W. 1st Boca Raton, FL, USA: CRC Press.

[B8] Chessa M. A. La Placa M. Patrizi A. Virdi A. Misciali C. Fedrizzi G. (2021). Chloracne: a case series on cutaneous expression of CYP1A1 as diagnostic biomarker. Clin. Exp. Dermatol. 46, 896–900. 10.1111/ced.14617 33638914

[B9] Clayton R. W. Gobel K. Niessen C. M. Paus R. van Steensel M. A. M. Lim X. (2019). Homeostasis of the sebaceous gland and mechanisms of acne pathogenesis. Br. J. Dermatol. 181, 677–690. 10.1111/bjd.17981 31056753

[B10] Connor K. T. Harris M. A. Edwards M. R. Budinsky R. A. Clark G. C. Chu A. C. (2008). Ah receptor agonist activity in human blood measured with a cell-based bioassay: evidence for naturally occurring ah receptor ligands *in vivo* . J. Expo. Sci. Environ. Epidemiol. 18, 369–380. 10.1038/sj.jes.7500607 17912254

[B11] Esteban J. Sanchez-Perez I. Hamscher G. Miettinen H. M. Korkalainen M. Viluksela M. (2021). Role of aryl hydrocarbon receptor (ahr) in overall retinoid metabolism: response comparisons to 2,3,7,8-tetrachlorodibenzo-p-dioxin (tcdd) exposure between wild-type and ahr knockout mice. Reprod. Toxicol. 101, 33–49. 10.1016/j.reprotox.2021.02.004 33607186

[B12] Feldman A. Mukha D. Maor I. I. Sedov E. Koren E. Yosefzon Y. (2019). Blimp1+ cells generate functional mouse sebaceous gland organoids *in vitro* . Nat. Commun. 10, 1, 10.1038/s41467-019-10261-6 31138796 PMC6538623

[B13] Fontao F. Barnes L. Kaya G. Sorg O. Saurat J. H. (2017). High susceptibility of LRIG1 sebaceous stem cells to TCDD in mice *Toxicol* . Sci 160, 230–243. 10.1093/toxsci/kfx179 28973660

[B14] Forrester A. R. Elias M. S. Woodward E. L. Graham M. Williams F. M. Reynolds N. J. (2014). Induction of a chloracne phenotype in an epidermal equivalent model by 2,3,7,8-tetrachlorodibenzo-p-dioxin (TCDD) is dependent on aryl hydrocarbon receptor activation and is not reproduced by aryl hydrocarbon receptor knock down. J. Dermatol. Sci. 73, 10–22. 10.1016/j.jdermsci.2013.09.001 24161567 PMC3885976

[B15] Furness S. G. Whelan F. (2009). The pleiotropy of dioxin toxicity--xenobiotic misappropriation of the aryl hydrocarbon receptor's alternative physiological roles. Pharmacol. Ther. 124, 336–353. 10.1016/j.pharmthera.2009.09.004 19781569

[B16] Gawkrodger D. J. Harris G. Bojar R. A. (2009). Chloracne in seven organic chemists exposed to novel polycyclic halogenated chemical compounds (triazoloquinoxalines). Br. J. Dermatol. 161, 939–943. 10.1111/j.1365-2133.2009.09302.x 19558551

[B17] Geueke A. Niemann C. (2021). Stem and progenitor cells in sebaceous gland development, homeostasis and pathologies. Exp. Dermatol. 30, 588–597. 10.1111/exd.14303 33599012

[B18] Geyer H. J. Scheunert I. Rapp K. Gebefugi I. Steinberg C. Kettrup A. (1993). The relevance of fat content in toxicity of lipophilic chemicals to terrestrial animals with special reference to dieldrin and 2,3,7,8-tetrachlorodibenzo-p-dioxin (TCDD). Ecotoxicol. Environ. Saf. 26, 45–60. 10.1006/eesa.1993.1040 7691535

[B19] Guyot E. Chevallier A. Barouki R. Coumoul X. (2013). The ahr twist: ligand-dependent ahr signaling and pharmaco-toxicological implications. Drug Discov. Today 18, 479–486. 10.1016/j.drudis.2012.11.014 23220635

[B20] Hahn M. E. (2002). Aryl hydrocarbon receptors: diversity and evolution. Chem. Biol. Interact. 141, 131–160. 10.1016/s0009-2797(02)00070-4 12213389

[B21] Harr T. French L. E. (2010). Toxic epidermal necrolysis and Stevens-Johnson syndrome. Orphanet J. Rare Dis. 5, 39–11. 10.1186/1750-1172-5-39 21162721 PMC3018455

[B22] Hayes J. D. Dinkova-Kostova A. T. McMahon M. (2009). Cross-talk between transcription factors ahr and nrf2: lessons for cancer chemoprevention from dioxin. Toxicol. Sci. 111, 199–201. 10.1093/toxsci/kfp168 19628587

[B23] Ikuta T. Ohba M. Zouboulis C. C. Fujii-Kuriyama Y. Kawajiri K. (2010). B lymphocyte-induced maturation protein 1 is a novel target gene of aryl hydrocarbon receptor. J. Dermatol. Sci. 58, 211–216. 10.1016/j.jdermsci.2010.04.003 20478695

[B24] Ju Q. Zouboulis C. C. (2016). Endocrine-disrupting chemicals and skin manifestations. Rev. Endocr. Metab. Disord. 17, 449–457. 10.1007/s11154-016-9371-2 27363826

[B25] Kim H. B. Um J. Y. Chung B. Y. Kim J. C. Kang S. Y. Park C. W. (2022). Aryl hydrocarbon receptors: evidence of therapeutic targets in chronic inflammatory skin diseases. Biomedicines 10, 1087. 10.3390/biomedicines10051087 35625824 PMC9139118

[B26] Kimbrough R. D. (1980). Human health effects of selected pesticides, chloroaniline derivatives. J. Environ. Sci. Health B 15, 977–992. 10.1080/03601238009372225 6449525

[B27] Ma C. Marlowe J. L. Puga A. (2009). The aryl hydrocarbon receptor at the crossroads of multiple signaling pathways. EXS 99, 231–257. 10.1007/978-3-7643-8336-7_9 19157064

[B28] McKee M. (2009). The poisoning of victor Yushchenko. Lancet 374, 1131–1132. 10.1016/S0140-6736(09)61027-8 19660808

[B29] Megna M. Napolitano M. Costa C. Balato N. Patruno C. (2017). Waste exposure and skin diseases. G. Ital. Dermatol Venereol. 152, 379–382. 10.23736/S0392-0488.17.05505-5 28209048

[B30] Niittynen M. Simanainen U. Syrjala P. Pohjanvirta R. Viluksela M. Tuomisto J. (2007). Differences in acute toxicity syndromes of 2,3,7,8-tetrachlorodibenzo-p-dioxin and 1,2,3,4,7,8-hexachlorodibenzo-p-dioxin in rats. Toxicology 235, 39–51. 10.1016/j.tox.2007.03.012 17448584

[B31] Ohtake F. Fujii-Kuriyama Y. Kawajiri K. Kato S. (2011). Cross-talk of dioxin and estrogen receptor signals through the ubiquitin system. J. Steroid Biochem. Mol. Biol. 127, 102–107. 10.1016/j.jsbmb.2011.03.007 21397018

[B32] Panteleyev A. A. Bickers D. R. (2006). Dioxin-induced chloracne--reconstructing the cellular and molecular mechanisms of a classic environmental disease. Exp. Dermatol. 15, 705–730. 10.1111/j.1600-0625.2006.00476.x 16881967

[B33] Park S. J. Park J. W. Park K. Y. Li K. Seo S. J. Kim B. J. (2021). Systemic contact dermatitis induced by rhus allergens in korea: exercising caution in the consumption of this nutritious food. Clin. Exp. Dermatol. 46, 324–327. 10.1111/ced.14458 32974941

[B34] Passarini B. Infusino S. D. Kasapi E. (2010). Chloracne: still cause for concern. Dermatology 221, 63–70. 10.1159/000290694 20516651

[B35] Puga A. Ma C. Marlowe J. L. (2009). The aryl hydrocarbon receptor cross-talks with multiple signal transduction pathways. Biochem. Pharmacol. 77, 713–722. 10.1016/j.bcp.2008.08.031 18817753 PMC2657192

[B36] Rudyak S. G. Usakin L. A. Tverye E. A. Orekhov A. S. Belushkina N. N. Paus R. (2018). Retinoic acid co-treatment aggravates severity of dioxin-induced skin lesions in hairless mice via induction of inflammatory response. Biochem. Biophys. Res. Commun. 506, 854–861. 10.1016/j.bbrc.2018.10.126 30389142

[B37] Ryan J. J. (2011). The Yushchenko dioxin poisoning: Chronology and pharmacokinetics. Hoboken, NY, USA: Wiley. A. Schecter.

[B38] Saurat J. H. Kaya G. Saxer-Sekulic N. Pardo B. Becker M. Fontao L. (2012). The cutaneous lesions of dioxin exposure: lessons from the poisoning of victor yushchenko. Toxicol. Sci. 125, 310–317. 10.1093/toxsci/kfr223 21998131

[B39] Scarisbrick D. A. Martin J. V. (1981). Biochemical changes associated with chloracne in workers exposed to tetrachlorazobenzene and tetrachlorazoxybenzene. J. Soc. Occup. Med. 31, 158–163. 10.1093/occmed/31.4.158 6460133

[B40] Schneider M. R. Paus R. (2010). Sebocytes, multifaceted epithelial cells: lipid production and holocrine secretion. Int. J. Biochem. Cell Biol. 42, 181–185. 10.1016/j.biocel.2009.11.017 19944183

[B41] Sorg O. (2014). AhR signalling and dioxin toxicity. Toxicol. Lett. 230, 225–233. 10.1016/j.toxlet.2013.10.039 24239782

[B42] Sorg O. Zennegg M. Schmid P. Fedosyuk R. Valikhnovskyi R. Gaide O. (2009). 2,3,7,8-Tetrachlorodibenzo-p-dioxin (tcdd) poisoning in victor yushchenko: identification and measurement of tcdd metabolites. Lancet 374, 1179–1185. 10.1016/S0140-6736(09)60912-0 19660807

[B43] Steele E. J. Bellett A. J. McCullagh P. J. Selinger B. (1990). Reappraisal of the findings on agent Orange by the Australian royal commission. Toxicol. Lett. 51, 261–268. 10.1016/0378-4274(90)90068-w 2187281

[B44] Thiess A. M. Frentzel-Beyme R. Link R. (1982). Mortality study of persons exposed to dioxin in a trichlorophenol-process accident that occurred in the BASF AG on November 17, 1953. Am. J. Ind. Med. 3, 179–189. 10.1002/ajim.4700030209 6215858

[B45] Tijet N. Boutros P. C. Moffat I. D. Okey A. B. Tuomisto J. Pohjanvirta R. (2006). Aryl hydrocarbon receptor regulates distinct dioxin-dependent and dioxin-independent gene batteries. Mol. Pharmacol. 69, 140–153. 10.1124/mol.105.018705 16214954

[B46] Um J. Y. Kim H. B. Kang S. Y. Son J. H. Chung B. Y. Park C. W. (2020). 2,3,7,8-Tetrachlorodibenzo-p-Dioxin regulates the expression of aryl hydrocarbon receptor-related factors and cytokines in peripheral blood mononuclear cells and CD4+ T cells from patients with atopic dermatitis and psoriasis. Ann. Dermatol. 32, 360–369. 10.5021/ad.2020.32.5.360 33911769 PMC7992582

[B47] Viard I. Wehrli P. Bullani R. Schneider P. Holler N. Salomon D. (1998). Inhibition of toxic epidermal necrolysis by blockade of CD95 with human intravenous immunoglobulin. Science 282, 490–493. 10.1126/science.282.5388.490 9774279

[B48] Zamarron A. Morel E. Lucena S. R. Mataix M. Perez-Davo A. Parrado C. (2019). Extract of Deschampsia Antarctica (EDA) prevents dermal cell damage induced by UV radiation and 2,3,7,8-Tetrachlorodibenzo-p-dioxin. Int. J. Mol. Sci. 20, 1356. 10.3390/ijms20061356 30889822 PMC6471785

