# Corrigendum: Development of skin diseases following systemic exposure: example of dioxins

**DOI:** 10.3389/ftox.2023.1323294

**Published:** 2023-12-22

**Authors:** Olivier Sorg, Jean-Hilaire Saurat

**Affiliations:** Clinical Pharmacology and Toxicology, University of Geneva, Geneva, Switzerland

**Keywords:** dioxin, skin, toxicology, AhR, biomarkers, ingestion

In the published article there was an error. In the **Abstract**, line 10, the sentence previously stated:

“Sweat glands release their lipid content on the surface of the skin by a holocrine secretion, and so any lost sebocyte should be transmitted to progenitor cells to differentiate and migrate to the sebaceous gland to replace the lost sebocyte.”

The corrected sentence appears below:

“Sebaceous glands release their lipid content on the surface of the skin by a holocrine secretion, and so any lost sebocyte should be transmitted to progenitor cells to differentiate and migrate to the sebaceous gland to replace the lost sebocyte.”

The authors apologize for this error and state that this does not change the scientific conclusions of the article in any way. The original article has been updated.

